# War and peace: exploring microbial defence systems as a source of new antimicrobial therapies

**DOI:** 10.3389/fphar.2024.1504901

**Published:** 2025-01-07

**Authors:** Paul J. Dyson, Ibrahim M. Banat, Gerry A. Quinn

**Affiliations:** ^1^ Medical School, Institute of Life Sciences, Swansea University, Swansea, United Kingdom; ^2^ Centre for Molecular Biosciences, Ulster University, Coleraine, United Kingdom

**Keywords:** antimicrobial resistance (AMR), antibiotic, WHO priority pathogens, sustainable antibiotic therapies, combination (combined) therapy

## Abstract

The WHO has compiled a list of pathogens that urgently require new antibiotics in response to the rising reports of antibiotic resistance and a diminished supply of new antibiotics. At the top of this list is fluoroquinolone-resistant *Salmonella typhi*, fluoroquinolone-resistant *Shigella* spp. and vancomycin-resistant *Enterococcus faecium*. Although these problems have been covered in great detail by other contemporary reviews, there are still some fundamental gaps in the translation of current knowledge of the infectious process and the molecular ecology of antibiotic production into a sustainable protocol for the treatment of pathogenic diseases. Therefore, in this narrative review we briefly discuss newly approved antimicrobial drugs (since 2014) that could help to alleviate the burden of multiresistant pathogens listed on the WHO priority list. Being conscious that such treatments may eventually run the risk of future cycles of resistance, we also discuss how new understandings in the molecular ecology of antibiotic production and the disease process can be harnessed to create a more sustainable solution for the treatment of pathogenic diseases.

## Introduction

The ancient Chinese military strategist Sun Tze famously wrote that “if you know your enemy and know yourself, you need not fear the result of a hundred battles”. Similarly, physicians have remained in the dark for many years about the real causes of pathogenic diseases and it was only when they started to discover the true nature of “the enemy” that progress began to be made. This was first seen in the field of antiseptics (Lister) and sterilization (Pasteur) which were able to eradicate many of the potentially pathogenic microorganisms in the clinical environment. Later, the discovery of penicillin and streptomycin ushered in the new era of antibiotics where pathogenic microorganisms could be directly confronted at the heart of the disease (Fleming, 1929; Schatz et al., 1944). Henri Waksman, another microbiologist and co-discoverer of streptomycin, later coined the term “antibiotics” to describe the molecules which antagonise the growth of pathogenic microbes (Ribeiro da Cunha et al., 2019). However, even with this great breakthrough, the understanding of the molecular ecology that surrounds the production of antibiotics was still in a nascent stage and scientists were somewhat surprised when resistance first arose to their wonder drug, penicillin (Davies and Davies, 2010). For many years after this, antimicrobial drug resistance did not constitute such a great problem because of the rapid turnover of new antibiotics. Of course, with the increase in reports of antibiotic resistance and the rapid slow-down in the discovery process in the 1970s, it became obvious that the existing antibiotic drug discovery platform which concentrated almost exclusively on one discovery methodology, a limited group of antibiotic producing organisms and a mono-therapeutic approach towards treatment, did not provide a sustainable solution to the problem of microbial infection and resistance (Ribeiro da Cunha et al., 2019). Although there was a brief transition period to a fast-throughput combinatorial discovery processes by designing new antibiotics from previous core structures, the return on investment for many pharmaceutical companies was uneconomical (Bartlett et al., 2013; Ribeiro da Cunha et al., 2019).

As a temporary solution to the problem of antibiotic resistance, many of the latest antibiotics are modifications of older discoveries, however, this means that resistance could develop far quicker (Davies and Davies, 2010). The holy grail of today’s antibiotic discovery is to identify compounds with new core structures or different modes of action from previous antibiotics so that the development of antimicrobial resistance will be delayed (Brüssow, 2024). These new structures are commonly referred to as “first in class”. Worryingly, only a few of these antibiotics have been approved in the last decade, causing many professional clinicians to warn that we are reaching crisis point again (Brüssow, 2024). Therefore, the world health organisation (WHO) has made a list of the top microbial pathogens for which antibiotics are urgently required. Even though there are many good in-depth reviews on this topic (Brüssow, 2024; Lombardi et al., 2024; Naghavi et al., 2024; Tängdén et al., 2024; Terreni et al., 2021), there are still major gaps in our understanding of what constitutes a genuinely sustainable treatment for pathogenic diseases. These gaps include an accurate assessment of antibiotic resistance *in-vivo* pathogenic diseases and the contribution of the environment from which they are isolated (Sodhi et al., 2023). Additionally, many assessments of therapeutic doses of antibiotics and treatments of microbial diseases are based on the planktonic growth form of pathogenic bacteria which are more susceptible to antibiotics rather than the higher doses needed to combat their biofilm counterparts (Anju et al., 2022). In addition, the WHO priority list specifies pathogens with specific resistance mechanisms, but it does not target the wild type of the microorganism *per se*, given the availability of treatment options. Most importantly, given that antibiotics and resistance elements have coexisted for millions of years, we have yet to see a successful translation of this molecular ecology into a sustainable form of antimicrobial chemotherapy.

Therefore, this narrative review discusses some of the latest additions to antimicrobial chemotherapy, considers the wider context of the molecular ecology of antibiotic production and examines these in the light of new understandings of the infection process. We then suggest how these new understandings might be applied to create a more sustainable form of antimicrobial chemotherapy.

## Methodology

This narrative review of recently published studies of antibiotic chemotherapies (since 2014) was carried out using PubMed and Google Scholar using the keywords, antibiotic resistance, WHO priority list, new antibiotics.

## Knowing the enemy, the WHO list of priority pathogens

Antibiotic resistance was observed not long after the discovery of penicillin and was indeed mentioned by Fleming during his Nobel prize acceptance speech (Abraham and Chain, 1940; Lobanovska and Pilla, 2017). This antibiotic resistance was compounded by a precipitous decline in the approval of new antibiotics since the 1970s (Ribeiro da Cunha et al., 2019). Indeed, a recent global analysis on the rise of antimicrobial resistance found that over a million people already died between 1990 and 2021 as a result of a drug-resistant infection. It is now predicted that a further 39 million more people could die from antibiotic-resistant infections between now and 2050 if immediate preventative action is not taken (Naghavi et al., 2024). On a positive note, it is estimated that 92 million lives could be saved if patients could have access to better treatment options (Naghavi et al., 2024).

Multiresistant bacteria are frequently classified into groups in the clinical environment. One of the most problematic groups of organisms found in the clinical environment are the ESKAPE pathogens, which include: *Enterococcus faecium, Staphylococcus aureus, Klebsiella pneumoniae, Acinetobacter baumannii, Pseudomonas aeruginosa,* and *Enterobacter* spp. (Santajit and Indrawattana, 2016). In more recent times, the WHO have created a list of pathogens for which new antibiotics are urgently needed in all environments. These pathogens are ranked by their resistance to treatment, prevalence, mortality rate, and the burden they place on the healthcare system (Tacconelli et al., 2018). Currently there are twenty four bacterial pathogen-drug combinations listed in this ranking which are further divided into combinations of “critical”, “high” and “medium” concern (Table 1). The top of this list is the *critical category* which includes infectious diseases which are difficult to prevent and are highly transmissible. These pathogens are generally known to have widespread mechanisms of resistance either at the global level and/or at local level in certain groups or distinct geographical areas. This group includes *Salmonella typhi*, *Shigella* spp. and *Enterococcus faecium.* The level below this is the high priority category which includes pathogens that are almost certainly difficult to treat and also have a substantial disease burden which is often reflected in the morbidity and mortality data. These pathogens are highly transmissible, having an increasing trend of resistance and are usually difficult to prevent. There are very few options for treatment of these pathogens but these are usually in the developmental stage. Although pathogens in this category may not be critical on a global scale, they could become a significant problem to some populations or in specific geographical areas. Some examples include methicillin resistant *Staphylococcus aureus* (MRSA) and *Enterobacteria.* The final group, “the medium category of antimicrobial resistant pathogens” are moderately difficult to treat, have a moderate resistance and have similar issues with prevention and transmission. There are often more treatment options for these pathogens, and they might not be of high importance on a global scale. However, they may cause significant problems at a local level or in selected sub-populations such as old people in nursing homes. This group also includes *Streptococcus pneumoniae* and *Haemophilus influenzae* (Tacconelli et al., 2018).

**TABLE 1:** WHO bacterial priority pathogens list, 2024.

Group	Organism
Critical group	*Salmonella typhi* fluoroquinolone-resistant
	*Shigella* spp. fluoroquinolone-resistant
	*Enterococcus faecium* vancomycin-resistant
	*Mycobacterium tuberculosis*, rifampicin-resistant aRR-TB was included after an independent analysis with parallel criteria and subsequent application of an adapted MCDA matrix
High group	*Pseudomonas aeruginosa* carbapenem-resistant
	Non-typhoidal *Salmonella* fluoroquinolone-resistant
	*Neisseria gonorrhoeae* third-generation cephalosporin, and/or fluoroquinolone-resistant
	*Staphylococcus aureus* methicillin-resistant
	*Enterobacterales* carbapenem-resistant
	*Enterobacterales* third-generation cephalosporin-resistant
	*Acinetobacter baumannii* carbapenem-resistant
Medium group	Group A Streptococci macrolide-resistant
	*Streptococcus pneumoniae* macrolide-resistant
	*Haemophilus influenzae* ampicillin-resistant
	*Group B Streptococci* penicillin-resistant
	Group A Streptococci macrolide-resistant

Gram-negative bacteria are often more resistant than Gram-positive bacteria to antibiotics because they have an outer membrane which is more impervious to penetration (Breijyeh et al., 2020). In addition, their outer membrane contains efflux pumps which can actively transport antibiotics out of their system. Therefore, there is a smaller pool of antibiotics available for Gram negative infections which in turn significantly increases the risk of resistance and creates a larger problem (Melander et al., 2023). The WHO priority pathogens list is frequently updated since the prevalence of the pathogen and antibiotic pairs is continually changing. Currently, the top of this list is fluoroquinolone-resistant *Salmonella typhi*, which can infect the human digestive system resulting in a high fever which can be fatal. In ordinary circumstances severe cases would be treated with fluoroquinolones, however, there are reports of resistant strains developing (Jun et al., 2018). The next pairing on the WHO list is fluoroquinolone resistant *Shigella* spp., which mainly affects young children in low income countries and can cause severe dysentery. The treatments options for this pathogen are usually beta-lactams or more commonly fluoroquinolones like ciprofloxacin. However, given current trends in resistance, treatment options are extremely limited (Kherroubi et al., 2024). The third pathogen, antibiotic pairing is vancomycin-resistant *E*. *faecium* which is normally part of the human microbial gut flora. However, it can also be associated with nosocomial infections of the urinary tract, abdomen and bloodstream. These pathogens can be treated with vancomycin but there are reports of resistant strains developing (Eichel et al., 2023).

## Do we have a good understanding of the enemy: unique challenges posed by microbial biofilms

Although the WHO has provided a list of priority pathogens for which antibiotics are urgently needed, most of these assessments have been made on planktonic growth form of bacteria, that is, free living, independent organisms. However, what many reviews of antimicrobial chemotherapy neglect to mention is that many persistent microbial infections are caused by microbial biofilms (Brüssow, 2024; Tängdén et al., 2024; Terreni et al., 2021). The biofilm physiology is significantly different from its planktonic counterpart and is characterised by a sessile, multicellular organisation of bacteria with cellular differentiation and internal architecture surrounded by a matrix of extracellular polymeric substances (EPS) composed of proteins, polysaccharides, extracellular DNA, extracellular enzymes and lipids (Anju et al., 2022). Although this might seem like an academic discussion point, biofilms can be 100–1,000 times more resistant to antibiotics than their planktonic counterparts of the same species (Olsen, 2015). Biofilms can be comprised of single bacterial species, mixed species and even include fungi and viruses (Anju et al., 2022). The resistance of microbial biofilms to antibiotics can be compounded by EPS production, which can reduce penetration or diffusion of the antibiotic and extracellular enzymes which can potentially break down antibiotics. Furthermore, the hydrophobic nature of the apical layer of the biofilm and the senescent nature of some biofilm cells (slow growing dormant cells) can render antibiotics ineffective (Banat et al., 2014). While cells within biofilms exhibit a much higher minimum inhibitory concentration of antibiotics, topical administration allows for delivery of elevated antibiotic concentrations (Anju et al., 2022).

## Knowing your strengths: new antibiotics against multiresistant pathogens

Antibiotics are chosen to inhibit unique bacterial physiological processes (so they do not affect human physiology) such as cell wall synthesis (beta-lactams), protein synthesis in the ribosomes (aminoglycosides, macrolides) or DNA/RNA transcription or translation (fluoroquinolones). Many of these antibiotics consist of a biologically active core or nucleus, surrounded by variable side-groups which may or may not be necessary to maintain this activity. Scientists have been chemically modifying these variable groups to create new variants which can overcome microbial resistance. Unfortunately, this strategy works both ways since small changes in the antibiotic target sites of pathogens or the enzymes they produce may also be enough to overcome the effectiveness of new chemotherapeutics (Ruef et al., 2024).

Currently the most concerning antibiotic resistance on the WHO priority list is to fluoroquinolones, carbapenems and glycopeptides such as vancomycin. Fluoroquinolones are broad spectrum antimicrobials like ciprofloxacin which target bacterial DNA gyrase and topoisomerase IV. Fluoroquinolones also interfere with supercoiling of DNA in the bacterial cell and can result in impaired DNA replication and cell death (Kherroubi et al., 2024). These antibiotics are recommended for multiresistant infections and hospital acquired pneumonia. Resistance to fluoroquinolones can be a major problem in the treatment of multidrug-resistant tuberculosis (MR-TB) and Gram-negative infections (Kherroubi et al., 2024; Nehru et al., 2024). This resistance is thought to arise through three key mechanisms: 1. Bacterial efflux pumps, 2. Production of protective proteins that bind to bacterial DNA gyrase or 3. Mutations in a key antibiotic binding site of DNA gyrase/topoisomerase which reduces antibiotic binding affinity (Kherroubi et al., 2024). The United Kingdom government recently recommended that fluoroquinolone antibiotics must now only be prescribed when other commonly recommended antibiotics are inappropriate.

Carbapenems are a sub-class of beta-lactams that contain a five-membered penicillin-like ring, however, the sulphur group at position C-1 is replaced by a carbon atom and a double bond between C-2 and C-3 is introduced. Carbapenems inhibit cell wall synthesis by attaching to penicillin-binding proteins (PBPs), enzymes that are essential for the final stages of peptidoglycan cross-linking. This prevents PBPs from catalyzing transpeptidation resulting in cell lysis and death (Kumar et al., 2023). Resistance to carbapenems is particularly concerning in relation to *A. baumannii, Enterobacterales and P. aeruginosa* infections. This resistance can stem from the production of carbapenemase (which can also be spread by transferable carbapenemase-encoding genes), increased expression of bacterial efflux pumps or decreased expression of porins (Naghavi et al., 2024).

Vancomycin is a cyclic glycosylated peptide and as such is part of the glycopeptide group of antimicrobials. This antibiotic inhibits the synthesis of peptidoglycan by binding to amino acids (d-alanyl-d-alanine) in the cell wall and preventing the addition of new units (Geraci et al., 1956). Vancomycin-resistant enterococci (VRE) infections usually occur in healthcare settings and are thought to be mediated through plasmids or transposons, although certain strains have natural resistance. Vancomycin resistance occurs when the terminal amino acid residues of peptidoglycan are changed preventing the antibiotic from binding to the cell wall (Eichel et al., 2023).

**FIGURE 1:**
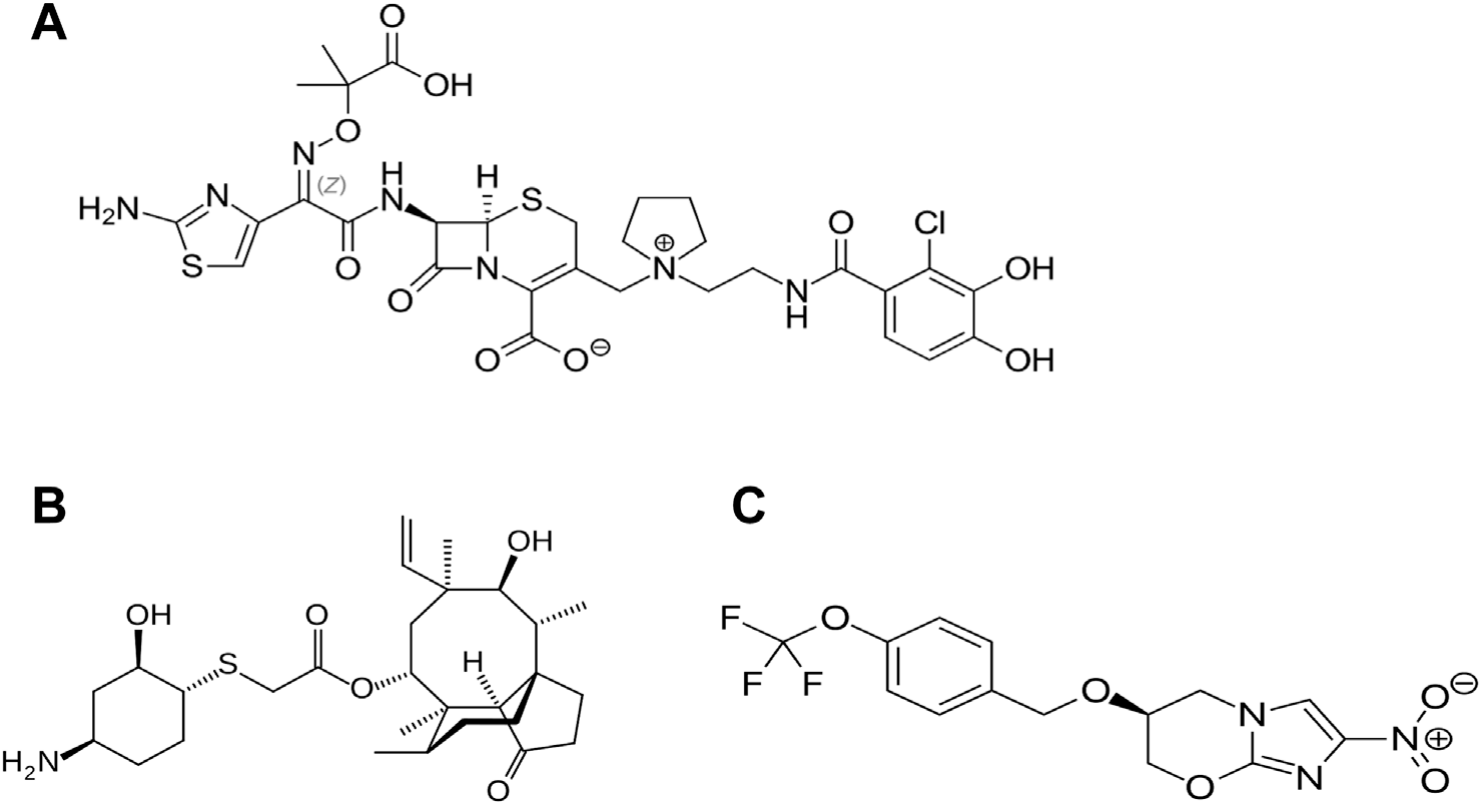
Recently approved “first in class” antibiotics, **(A)** cefiderocol, **(B)** lefamulin and **(C)** pretomanid. Image source: Creative Commons CC0 1.0 Universal Public Domain Dedication.

Several new candidates have been proposed as antibiotics to treat infections for multi-resistant organisms (since 2014); however, most of these are based on previous antibiotic core structures, such as fluoroquinolones. Worryingly there are only three new “first in class” that have been approved since this time. The first of these is cefiderocol (Figure 1A), a new injectable, siderophore/cephalosporin antibiotic which was approved in 2019 by the FDA for the treatment of Gram-negative pathogens especially in cases of urinary tract infection (UTI) or hospital acquired pneumonia (HAP)/ventilator acquired pneumonia (VAP) in 2020 (Wu et al., 2020). The novelty of this antibiotic/siderophore combination is that it enters through the outer membrane of Gram-negative pathogens attached to the bacteria’s iron uptake system (siderophore). This mode of entry achieves higher concentrations of antibiotic in the periplasmic space where the cephalosporin can bind to the penicillin binding proteins (PBP) and inhibit cell wall synthesis. The inhibitory spectrum of these antibiotics includes carbapenem-resistant Enterobacterales (CRE) that produce serine- and/or metallo-carbapenemases, multi-resistant *Acinetobacter baumannii*, *P. aeruginosa*, *Stenotrophomonas maltophilia*, *Achromobacter* spp. and *Burkholderia* spp. Note, cefiderocol also has a warning label for higher all-cause mortality in relation to other antibiotics in critically ill patients with multidrug-resistant Gram-negative bacterial infections due to currently unexplained effects (Viale et al., 2023; Wu et al., 2020).

**FIGURE 2:**
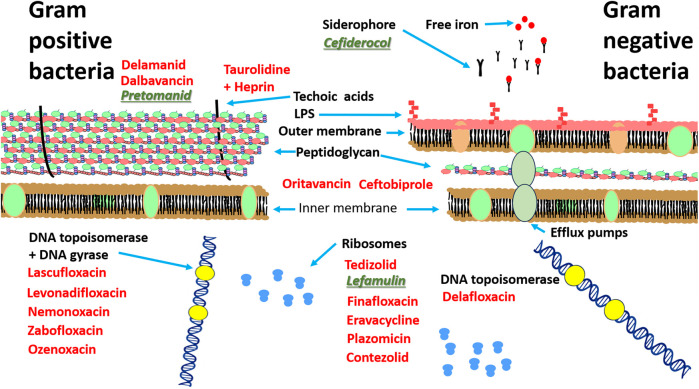
Site of action of antibiotics approved since 2014 against Gram-positive and Gram-negative bacteria. Antibiotics based on known structures and activity are highlighted in red, “first in class” antibiotics are highlighted in green.

Lefamulin is another of the “first in class” antibiotics (Figure 1B). This is a pleuromutilin derivative which was approved for treatment of community-acquired bacterial pneumonia (CABP) by FDA in 2019. Its mechanism of action involves the inhibition of protein synthesis by preventing the binding of transfer RNA for peptide transfer. This antibiotic has shown inhibitory activity against *Streptococcus pneumoniae*, *Haemophilus influenzae*, *Moraxella catarrhalis, Legionella pneumophila, Mycoplasma pneumoniae* and *Chlamydophila pneumoniae* (Zhanel et al., 2021). The third “first in class” antibiotic is pretomanid (Figure 1C) which is used against highly resistant strains of *Mycobacterium tuberculosis* including rifampicin resistance. Its mode of action involves the inhibition mycolic acid in the bacterial cell wall (Gils et al., 2022). A list of the other antibiotics that have approved since 2014 is provided in Table 2 and the approximate sites of their mode of action in Figure 2.

**TABLE 2:** Newly approved antibiotics (since 2014) that are effective against multi-resistant bacteria.

Antibiotic	Approval	Class	Condition	Inhibits	Ref
Cefiderocol	2019 United States, first in class	Siderophore + cephalosporin	HABP VABP UTI	*A. baumannii*, CRE	Wu et al. (2020)
Contezolid	2021 China	Oxazolidinone	cSSTI	MRSA, MSSA, *Streptococcus pyogenes*	Li et al. (2023)
Ceftobiprole	2024 United States	Cephalosporin	HABP CABP VABP	*P. aeruginosa* and *A. baumannii*	Li et al. (2024)
Dalbavancin	2014 United States	Lipoglycopeptide	ABSSSI	MRSA	
Delafloxacin	2017 United States	Fluoroquinolone	ABSSSI CABP	Biofilms MRSA, *K. pneumoniae, Legionella pneumophila*	Craddock et al. (2023)
Delamanid	2014 Europe	Nitroimidazole	TB	MDR-TB	Schönfeld et al. (2024)
Finafloxacin	2014 United States	Fluoroquinolone	acute otitis externa	MRSA, quinolone-resistant MRSA, *Yersinia pestis*	Kocsis et al. (2021)
Lascufloxacin	2019 Japan	Fluoroquinolone	ENT, CAP	Fluoroquinolone resistant respiratory pathogens	Shimada and Seki (2024)
Lefamulin	2019 United States, first in class	Pleuromutilin derivative	CABP	MRSA, VRSA, hVISA	Zhanel et al. (2021)
Levonadifloxacin	2019 India	Fluoroquinolone	ABSSSI	MRSA, quinolone-resistant *S. aureus*, macrolide- and penicillin-resistant *S*. *pneumoniae*. Biofilms	Bhagwat et al. (2019)
Nemonoxacin	Russia, Turkey China	Non-fluorinated quinolone	CAP	*S. pneumoniae*, *Staphylococcus aureus and* MRSA.	Huang et al. (2022)
Eravacycline	2018 United States	Tetracycline	cIAI	Gram-positive, Gram-negative, Mycobacteria	Kunz et al. (2023)
Ozenoxacin (xepi)	2017 United States	Quinilone	ABSSSI	MRSA isolates non-susceptible to ciprofloxacin	García-Castillo et al. (2024)
Oritavancin	2014 United States	Tetracycline	ABSSSI	MRSA	Brade et al. (2016)
Plazomicin (zemdri)	2018 United States	Aminoglycoside	cUTI. VABP, pyelonephritis	Multi-resistant *E. coli*, *K. pneumoniae*, *A. baumannii*, *P. aeruginosa*, *S. aureus and* CRE	Alfieri et al. (2022)
Pretomanid	2019 United States, first in class	Nitroimidazole	TB	MDR-TB	Gils et al. (2022)
Tedizolid (sivextro)	2014 United States	Oxazolidinone	ABSSSI	Gram-positive, MRSA	Salavert Lletí et al. (2021)
Taurolidine + heparin (defencath)	2023 United States	Amino acid	UTI, catheter-related infections	MRSA, VRE	Agarwal et al. (2023)
Zabofloxacin	2015 South Korea	Non-fluorinated quinolone	AECOPD	Drug-resistant *Neisseria gonorrhoeae* and *Streptococcus pneumoniae* and MRSA	Kocsis et al. (2021)

Abbreviations: hospital associated bacterial pneumonia (HABP), ventilator associated bacterial pneumonia (VABP), complicated intra-abdominal infection (cIAI), complicated skin and soft tissue infection (cSSTI), urinary tract infection (UTI), community acquired bacterial pneumonia (CABP), acute bacterial skin and skin structure infections (ABSSSI), acute exacerbation of chronic obstructive pulmonary disease (AECOPD), multi-drug resistant TB (MDR-TB).

There are also other “first in class” antibiotics that are still in the testing stages, the most promising candidates of these so far are gepotidacin, odilorhabdin and zosurabalpin. Gepotidacin is an antibiotic discovered in 2007 at Glaxo-Smith-Kline (GSK) but is still at the testing stages. It is recommended for UTIs and resistant strains of *N. gonorrhoeae* (Ali and Anderson, 2024). Its mechanism of action involves the inhibition of two topoisomerase enzymes and induction of single-stranded breaks in the bacterial DNA (Ali and Anderson, 2024). It is effective against *S. aureus,* MRSA, *Streptococcus pneumoniae* (including penicillin-nonsusceptible isolates) and *E. coli* (Flamm R. K. et al., 2017). In research trials, the “MIC_90_ of gepotidacin for fifty isolates of *S. aureus* (including MRSA) and fifty isolates of *S. pneumoniae* (including penicillin-nonsusceptible) was 0.5 μg/mL, and for *E. coli* (*n* = 25 isolates), it was 4 μg/mL” (Flamm R. K. et al., 2017). Although administration of gepotidacin is generally well tolerated in patients with acute bacterial skin and skin structure infections (ABSSSI) in trials, there are still adverse events (AE) in approximately 40% of trial participants, most frequently reported were nausea (20%) and diarrhoea (13%) but the majority of AE (51%) were mild. In addition, gepotidacin at a 1,000-mg dose is already known to lead to a corrected QT interval (QTcF) prolongation of approximately 12 ms (O’Riordan et al., 2017).

Odilorhabdin was another much heralded “first in class” antibiotic, whose discovery was announced more than 10 years ago in 2013, however the website of the company managing this antibiotic shows that this therapy has not yet entered phase 1 trials. This antibiotic has shown activity against Gram-positive and Gram-negative pathogens in research studies, including carbapenem-resistant *Enterobacteriaceae* (CRE). Its mechanism of action is based on binding to the small ribosomal subunit at a site not exploited by current antibiotics. This induces miscoding and promotes hungry codon readthrough, amino acid misincorporation, and premature stop codon bypass (Pantel et al., 2018). In research studies of Odilorhabdin, a variety named NOSO-502 had an MIC value ranging from 0.5 to 4 μg/mL against standard *Enterobacteriaceae* strains and carbapenem-resistant *Enterobacteriaceae* (CRE) isolates (that produce KPC, AmpC, or OXA enzymes and metallo-β-lactamases) (Emilie et al., 2018). Studies also found that there was no cytotoxicity against the cell lines “HepG2, HK-2, human renal proximal tubular epithelial cells (HRPTEpiC), hERG-CHO or Nav 1.5-HEK current, and no increase of micronuclei at 512 μM” (Emilie et al., 2018).

Finally, Zosurabalpin is another “first in class” antibiotic which is still in Phase I trials. This has been shown to be effective against carbapenem-resistant *Acinetobacter baumanii* (CARB) in research studies and would be a welcome addition to the clinical pharmacy since no new class of antibiotic with activity against this bacteria has been approved for the last 50 years. The main mechanism of this drugs activity is the inhibition of the ATP-binding cassette transporter lipopolysaccharide transporter complex (LptB_2_FG) that assembles the outer membrane of Gram-negative bacteria (Zampaloni et al., 2024). It was reported that using the Clinical Laboratory Standards Institute (CLSI) broth dilution method, “zosurabalpin was active against *Acinetobacter* spp., with an MIC_50/90_ of 0.12/0.5 μg/mL and 0.25/1 μg/mL in cation-adjusted Mueller Hinton broth (CA-MHB) supplemented with 20% of horse serum and human serum (HS), respectively (MIC range of 0.015/0.03–8 μg/mL)” (Hawser et al., 2023). At the present moment research reports indicate that zosurabalpin is non-cytotoxic (Zampaloni et al., 2024).

The combination of several pharmaceutical drugs is also a well-known method of preventing bacterial resistance. The best known example of this is probably in *Mycobacterium tuberculosis* infections where several antibiotic compounds have been administered for many decades, i.e., isoniazid in combination with ethambutol, rifampin or pyrazinamide (Rabahi et al., 2017). In addition, antibiotics can be combined with corresponding bacterial resistance inhibitors, such as a beta-lactamase inhibitor in combination with a beta-lactam as is the case for clavulanic acid and amoxicillin. Recently approved combinations of similar compounds include:

Ceftolozane/Tazobactam (zerbexa). This combination was approved by FDA in 2014 for urinary tract infections (UTIs), intra-abdominal infections (IAI) and later in 2019 for hospital associated bacterial pneumonia and ventilator associated bacterial pneumonia (HABP/VABP). This combination is used against extended-spectrum beta-lactamases ESBL but some resistant mutants have been already described. Possible adverse events to this combination includes nausea and diarrhoea (López Montesinos et al., 2021).

Ceftazidime-avibactam (avycaz) was approved by the FDA in 2015 for complicated intra-abdominal infections (cIAI), later extended to VABP/HABP (Sanz Herrero, 2022). This combination of an approved cephalosporin (ceftazidime) and a novel β-lactamase inhibitor (avibactam) is effective against multidrug-resistant Gram-negative infections especially *Enterobacteriaceae*, including ceftazidime-resistant strains. As a side-note this combination is more likely to cause serious adverse events than meropenem (Yusuf et al., 2021).

Meropenem/Vaborbactam was approved by FDA in 2017 for the treatment of UTIs, IAIs and HABP. It is mainly effective against CRE (Duda-Madej et al., 2023; Yusuf et al., 2021).

Imipenem-Cilastatin/Relebactam (recarbrio) was approved by FDA in 2019. This combination is used against UTIs, IAI, HABP and VABP (Heo, 2021). It is recommended for the treatment of multidrug-resistant Gram-negative infections (Mansour et al., 2021). It also restores antimicrobial activity against *K. pneumoniae* isolates that harbour KPCs (Heo, 2021).

A variation on the theme of using several compounds to overcome resistance is to use antibiotics that can affect several different molecular targets at once such as macrolones, a novel class of macrolide antibiotics. These are dual action antibiotics are based on the conjugation of a macrolide and a quinolone side-chain. Their mechanism of action involves targeting the bacterial ribosome and DNA gyrase and can evade resistance mechanisms. Macrolones are characterized by low to moderate systemic clearance, a large volume of distribution, a long half-life, and low oral bioavailability. They are very effective against *Streptococcus pneumonia* without activating resistance genes (Aleksandrova et al., 2024).

## Replenishing the armoury: research and development into new sources of antibiotics

Of course, researchers need to constantly search for new sources of antibiotics to replenish the discovery pipeline. One of the least intensive methods from a methodological point of view is to analyse the whole genome sequence of a suspected antibiotic producing microorganism in a technique sometimes referred to as genome mining (Adamek et al., 2017). There are several databased containing the sequence information of known antibiotic gene synthesis clusters such as antiSMASH or PRISM, that can then be used on newly sequenced genomes to identify potential antibiotic clusters (Medema et al., 2011). Increasingly, genome mining can also be used to assess the spread of antibiotic resistance (Sodhi et al., 2023).

In addition to normal software programmes, researchers have also employed artificial intelligence (AI) systems to find new antibiotics to combat multidrug resistant pathogens. In a recent example, a new class of antibiotic effective against MRSA was identified using deep learning models (Wong et al., 2024). The researchers created a training data set which included almost 39,000 compounds which were evaluated for their antibiotic activity against MRSA. To refine their search parameters the researchers employed three deep learning models which assessed the toxicity of each compound on three different types of human cells. The team then identified structural based motifs that were connected to antimicrobial activity and ranked these to find the best candidates with the highest antibiotic activity and the lowest cytotoxicity. This model was then used to screen a library of 12 million compounds before arriving at 280 candidates for *in-vitro* testing. Filtering these results produced two promising candidates, one for skin infections and one for systemic infections which were then tested on mice. One of these compounds was inhibitory to MRSA and VRSA, evaded substantial resistance and reduced bacterial titres in mouse models of MRSA skin and systemic thigh infection (Wong et al., 2024).

Another source of new antibiotics is repurposed drugs that have already been approved for other clinical purposes. Repurposed drugs can reduce the concerns about safety risks and save time and money (Schcolnik-Cabrera et al., 2021). Ciclopirox, a topical antifungal agent which has been in use for 20 years now, has recently been identified as having antimicrobial activity against antibiotic-resistant Gram-negative bacteria such as *A. baumannii*, *E. coli*, and *K. pneumoniae.* This drugs mechanism of action involves interference in galactose metabolism of bacteria, the inhibition of the synthesis of lipopolysaccharide (LPS) and iron chelation (Carlson-Banning et al., 2013). Interestingly, new research suggests that combining ciclopirox with polymyxin B can modulate the resistance of multi-drug resistance of *E. coli* and *A. baumannii*, possibly allowing for reduced dosages of polymyxin B in treating infections by Gram-negative pathogens (Kim et al., 2015).

Berberine, an isoquinoline quaternary alkaloid derived from various medicinal plants which is currently in use as an anti-diarrhoea drug, has also demonstrated significant potential as an antibiotic adjuvant against multi-drug resistant bacteria and a promising candidate for combination therapy. It has recently demonstrated efficacy against multidrug-resistant *Mycobacterium tuberculosis* and MRSA (Zhou et al., 2023).

Niclosamide is a halogenated salicylanilide, that has been used as an anthelminthic drug. Recent repurposing research has shown that it also inhibits growth of *S. aureus*. Perhaps more importantly, niclosamide synergizes with colistin to reverse colistin resistance in Gram-negative bacteria. It has also been found to inhibit quorum sensing, leading to the subsequent inhibition of virulence factors and biofilm formation in *P. aeruginosa* (Domalaon et al., 2019; Zhang et al., 2022).

Anticancer drugs have also been repurposed as antibiotics such as mitomycin C (MMC) which has activity against opportunistic pathogens that causes severe infections, stationary-phase, persister, and biofilm cells. It is particularly effective against *E. coli*, *S. aureus*, *P. aeruginosa*, imipenem-resistant *K. pneumoniae* and *Borrelia burgdorferi*, the causative agent of Lyme disease. The antibacterial activity of MMC is enhanced against multiresistant Gram-negative bacteria when it is combined with tobramycin-ciprofloxacin hybrid (TOB-CIP) (Svedholm et al., 2024). Another anticancer medicines is 5-fluorouracil (5-FU) (Sharma et al., 2020) which has been successfully applied as an anti-infective external coating of central venous catheters in a randomized trial which compares it against chlorhexidine/silver sulfadiazine (Walz et al., 2010).

Even antipsychotic drugs have now been suggested as new antibiotics such as diphenylbutylpiperidine. In this case the antipsychotics were successfully used against *M. tuberculosis* and *Salmonella enterica* infections (Heemskerk et al., 2021).

Anti-inflammatory compounds have also been repurposed such as BAY 11–7,082. This has been shown to inhibit the growth of Gram-negative pathogens like *P. aeruginosa* and MRSA (Coles et al., 2022).

Some groups have combined a computational approach based on virtual screening of ligands (LBVS) with an experimental confirmation method to screen known bioactive compounds such as antibiotics (Molina-Panadero et al., 2024). Using two anticancer drugs as chemical templates for antibiotic activity, the group used topological fingerprinting to select twelve chemically diverse compounds to be screened as antimicrobials. This search was narrowed down to three thiophene derivatives with promising antibacterial activity against colistin resistant *A. baumannii* and *E. coli*. Although the antibacterial mechanism of action of these thiophenes is still unknown, research points towards further investigation of the bacterial outer membrane proteins. However, the researchers added a note of caution since they observed the appearance of colistin-resistant *A. baumannii* and colistin-resistant *E. coli* strains after treatment with some thiophene derivatives. However, this does not preclude this class of compound from some role in therapies against multi-resistant gram negative organisms (Molina-Panadero et al., 2024).

Another source of potential new antibiotics for the treatment of multiresistant pathogens are microorganisms isolated from extreme and unusual environments (Quinn and Dyson, 2024). It is thought that antibiotic producing bacteria from areas of high physiological stress or other unusual environments can have a significantly different antibiotic production systems than their mesophilic counterparts (Chen et al., 2022). As a consequence of this, it is assumed that they might produce an equally exotic repertoire of antibiotics or at least express different methods of antibiotic production. These unusual environments include areas of high physiological stress (deserts, artic), associations of antibiotic producers with animals or plants or antibiotic producing organisms isolated from areas that are associated with historic or traditional medicines (Liu et al., 2014; Müller et al., 2004; Quinn et al., 2020).

Extremely arid environments have proved to be a good resource of new antibiotics that can combat multiresistant organisms (Abdelkader et al., 2018; Masand et al., 2018). Among the discoveries that relate to multiresistant pathogens on the WHO priority list, a group of antibiotics designated Chaxamycins were discovered in the Atacama desert and have been reported to inhibit the growth of MRSA. Another group of antibiotics from the same geographical location named Chaxalactins have also been shown to inhibit the growth of *S. aureus* (Rateb et al., 2011).

At the other end of the physiological spectrum there are areas of extreme cold such as the polar ice-caps and the frozen tundra which have also yielded some useful discoveries. Noteworthy among these are *Lindgomycetaceae,* a group of fungi that are responsible for the production of lindgomycin, an antibiotic effective against *S. aureus*, *S. epidermidis* and methicillin-resistant *S. epidermidis* (MRSE) (B. Wu et al., 2015). Another fungal isolate isolated from cold seawater in the Barents sea, *Aspergillus protuberus* MUT3638 produces bisvertinolone, a member of sorbicillonoid family. This antibiotic is effective against *S. aureus* with a minimum inhibitory concentration (MIC) of 30 μg/mL (Corral et al., 2018). In addition there is also dixiamycin, purified from *Streptomyces olivaceus* OUCLQ19-3 isolated from a *cold seep* in the South China Sea. This antibiotic demonstrates good inhibitory activity against *Salmonella typhimurium* CCARM 8250, *S*. *aureus* CCARM 3090, *E*. *faecium* CCARM 5203 and *Enterococcus faecalis* CCARM 5172 (Jin et al., 2021). Another group of antibiotics, the phocoenamicins B and C were isolated from *Micromonospora sp.* which are classed as rare actinomycetes. These were identified from a sample of marine cave sediment in Gran Canaria (Spain) and belong to the spirotetronate class of polyketides. These antibiotics are effective against both MRSA and *Mycobacterium tuberculosis* H37Ra but they have no significant activity against vancomycin-resistant *Enterococcus* (VRE) (Pérez-Bonilla et al., 2018).

Another new or rediscovered source of antibiotics which may have a potential to combat multiresistant pathogens are *Streptomyces* associated with historical and traditional medicines. Of course, various plant and animal components have been used in historical medicines since the times of the Pharaohs to cure diseases (Metwaly et al., 2021). However, we doubt that knowledge of specific antibacterial components was well understood at this time. One of the first researchers in the modern era to investigate these traditional and historical medicines was Julian Davies and his research group in Canada (Behroozian et al., 2016). They tested a sample of local “healing” Kisameet clay from British Columbia that had been used for millennia by the Helsuit indigenous people which actively inhibited the growth of all six ESKAPE pathogens (Behroozian et al., 2016). The pathogens such as *E. faecium* and *S. aureus* strains exhibited resistance to carbapenems, first-generation cephalosporins, quinolones, tetracyclines, nitrofurantoin, clindamycin, and erythromycin. All Gram-negative strains were resistant to first- and second-generation cephalosporins and penicillin’s. In addition, *K. pneumoniae*, *A. baumannii*, and *P. aeruginosa* strains exhibited resistance to third-generation cephalosporins and trimethoprim (Behroozian et al., 2016). Remarkably, the presence of Kisameet clay dramatically reduced the viability of all strains tested. For example, there were no viable cells of *A. baumannii* and *Enterobacter* sp. after 5 h exposure to Kisameet clay. Additionally, *S. aureus, K. pneumoniae, P. aeruginosa, A. baumannii* AB-1264, and *Enterobacter cloacae* 1,172 lost viability completely after 24 h. Strains of *E. faecium* strains took slightly longer at 48 h (Behroozian et al., 2016). Although there are differences in susceptibility between isolates of the same species, to date no resistance to Kisameet clay has been observed. The Heiltsuk nation employ Kisameet clay in geophagia for a variety of internal ailments, suggesting that this clay might be an option for treatment of intractable infections such as *Clostridium difficile* (Behroozian et al., 2016). In addition the clay was also an extremely good source of *Streptomyces* isolates (Svensson et al., 2017).

Working on a similar theme, a group from Wales, examined clay from ancient Irish healing cure (Quinn et al., 2021; Terra et al., 2018) where they identified eight isolates of *Streptomyces* including *Streptomyces* sp. myrophorea which inhibited the growth of many strains of ESKAPE pathogens; most notably carbapenem-resistant *Acinetobacter baumannii*, vancomycin-resistant *E*. *faecium*, and methicillin-resistant *Staphylococcus aureus*. Additional genome sequencing identified 45 secondary metabolite biosynthetic clusters (Terra et al., 2018). The other seven isolates were also effective to varying degrees against several multiresistant pathogens and fungi (Quinn et al., 2021).

## Do we understand the war: new discoveries in molecular systems of antibiotic production

Another approach to developing technologies that can overcome multidrug resistance is to understand the physiology of the antibiotic production process in the context of its natural environment. As a caveat, we also understand that the small molecules that we call antibiotics may also have other functions such as signalling. That said, antibiotics are routinely produced by some microorganism when they mature, or in the presence of the appropriate cues. If the events surrounding the production of antibiotics under many conditions (both laboratory and environmental) are studied closely at a molecular level, it might be possible to increase our understanding about the process of antibiotic production and hence potential treatments. This is especially important in the discovery of new antibiotics from silent or cryptic antibiotic gene synthesis clusters or even to improve the expression of existing antibiotics. This is essentially the case for researchers who identified physiological differences in antibiotic production in a species of desert *streptomyces* named *S. violaceusniger* SPC6. After whole genome sequencing, the researchers noted that the bacteria’s genome contained a novel tRNA gene encoding tRNA-Asp-AUC. The complementary codon sequence to this on the reciprocal mRNA is GAT, which can be over-represented in pathways which result in the synthesis of antibiotics. The translation of this codon (GAT), in non-extreme or mesophilic *Streptomyces* species is usually subject to an inefficient wobble base pairing by the conserved tRNA-Asp-GUC. However, cloning and expression of this new tRNA in mesophilic *Streptomyces* that normally produce antibiotics resulted in their over-production. Most interestingly, from the point of view of identifying new antibiotics, this new tRNA was also responsible for the expression of silent or cryptic antibiotic gene synthesis clusters in *S. coelicolor*. Although it is not known at this stage whether this expression of silent gene clusters may extend to other organisms, it shows great potential for inducing the expression of new antibiotics in different species (Chen et al., 2022).

## Allies in the war: secondary metabolites as adjuvants in treatment of multiresistant pathogens

As discussed earlier, if we repeat the chemotherapeutic strategies of the past, i.e., treating infections with a single antibiotic, we might end up with the same problem of bacterial resistance further down the line. Historically, antimicrobial chemotherapies have been predicated on the administration of usually one antibiotic, however this is rarely the case in the natural environment. Antibiotics are just one of the many compounds produced by some organisms as part of larger repertoire of secondary metabolites. These are usually synthesised at the mature stage of growth, although some can be produced at any stage and can include compounds such as pigments, antioxidants, metal scavenging compounds and biosurfactants (Mattingly et al., 2020).

Originally, the premise of antibiotic purification was to screen out all the potential pyrogens or compounds that can cause fever, induce inflammatory cytokines or be potentially toxic (Lobanovska and Pilla, 2017). However, this purification process also removes other secondary metabolites which may be complementary to the activity and stability of the antibiotic. Indeed, recent research points to the fact that secondary metabolites can act as adjuvant compounds to some antibiotics (Kumar et al., 2023), that is, they might be helpful in the stability and enhancement of the activity of the main antibiotics. Most importantly for this review, research has shown that the addition of supplementary compounds such as adjuvants to the main antibiotic principle can also delay the onset of antimicrobial resistance (Dhanda et al., 2023; González-Bello, 2017; Mattingly et al., 2020).

Bacteria can transition from planktonic growth to biofilm growth under the influence of environmental conditions and nutrient availability. Interestingly this transition is sometimes aided by the release of another secondary metabolite known as a biosurfactant. Indeed, several recent solutions to the problem of bacterial infections and antibiotic resistance have proposed the use of biosurfactants as adjuvants for antibiotics (Banat et al., 2014; Thakur et al., 2024). The presumed mode of action of these compounds is to increase the permeability of bacterial cells and aid the delivery of the antibiotics to their target. Indeed, researchers have shown that combining biosurfactants with standard antimicrobials such as chlorhexidine, sodium lauryl sulphate, tetracycline and ciprofloxacin can lower their minimum inhibitory concentrations (Elshikh et al., 2017).

Biosurfactants are usually classified according to their hydrophilic moiety, e.g., rhamnolipids consist of a rhamnose sugar (hydrophilic) and a lipid tail (hydrophobic) while sophorolipids have a sophorose sugar linked to the lipidic tail (Elshikh et al., 2017). Sophorolipids, are a group of glycolipid biosurfactants derived from non-pathogenic yeasts which in recent years have been investigated to assess their potential as adjuvants to combat multi-drug resistance. Indeed it has been demonstrated that when combined with regular antibiotics such as tetracycline, they can increase the overall inhibitory activity against bacteria such as *S. aureus* by 25% (Joshi-Navare and Prabhune, 2013). Another anionic glycolipoprotein produced by *Lactiplantibacillus plantarum strain* 1,625 demonstrated strong antibacterial and antibiofilm characteristics against pathogenic strains such as *S. aureus* MTCC 1049 (Thakur et al., 2024). As discussed earlier biosurfactants can be especially useful in the treatment of microbial biofilms (Banat et al., 2014; Satpute et al., 2018). Research has shown that the addition of the biosurfactant, surfactin significantly prevented *S. aureus* biofilm formation in the case of diabetic foot ulcers and displayed limited toxicity on human red blood cells. Surfactin also demonstrates synergy with ampicillin, oxacillin and tetracycline against MRSA and did not readily produce *in vitro* resistance (Li Z. et al., 2023).

Other commonly produced secondary metabolites with adjuvant activity to antibiotics are antioxidants. Their potential adjuvant activity was not fully appreciated until research on human beta-defensin (HBD)-1 demonstrated that under the influence of thioredoxin (an antioxidant), HBD-1 could transform into a much more potent antibiotic that was capable of inhibiting the growth of MRSA, a pathogen on WHO priority list (Jaeger et al., 2013). Other antioxidants, such as alkylresorcinol DB-2073 can have antibiotic activity on their own but when combined with antibiotics such as vancomycin, gentamicin, polymyxin, ampicillin, can inhibit various pathogenic bacteria (Nikolaev et al., 2020).

Bacteria frequently use metal scavenging compounds or metal chelators/siderophores to supplement their iron/metal requirements as nutrients. However, these have only recently come to be discussed as antimicrobial adjuvants. In some cases these can also be used to deliver antibiotics to bacteria, which may help treat infections caused by antibiotic-resistant bacteria (Rayner et al., 2023) or potentially reinvigorate older antibiotics as discussed for berberine and niclosamide (Zhang et al., 2022; Zhou et al., 2023). Antibiotics can also be chemically linked to siderophores to ensure antibiotic delivery directly into cells which is a very important breakthrough in terms of Gram-negative resistance to antibiotics (Rodríguez and González-Bello, 2023). For example, desferrioxamine, produced by *Streptomyces pilosus* has synergistic activity with gentamicin, chloramphenicol, cefalothin, cefotiam and cefsulodin and bedaquiline against pathogenic bacteria (Cahill et al., 2021). Surprisingly, these siderophores have also been identified as naturally conjugated with antibiotics in nature in compounds known as siderophore–antibiotic conjugates (SACs) or sideromycins (Nüesch and Knüsel, 1967).

One of these groups of sideromycins are known as albomycins. These are produced by *Streptomyces griseus* and consist of a tri-hydroxamate iron chelating component, N-acetyl-N-hydroxy-L-ornithine, attached to an antibacterial thioribosyl pyrimidine moiety. This combination has very good inhibitory activity against both Gram-negative *E. coli*, and Gram-positive bacteria like *S. pneumoniae* including multi-drug resistant strains (Lin et al., 2018; Wang et al., 2022).

Another group of sideromycins, ferrimycins are a group of iron-containing siderophores (ferrioxamine B) attached to an antibiotic. These compounds have been investigated as a template for a “trojan horse” antibiotic delivery strategy. In one specific example researchers coupled pyridomycin to a chlorocatechol-containing siderophore named chlorodactyloferrin (Caradec et al., 2023). As described previously, this strategy is very important because Gram-negative bacteria have a siderophore receptor which may also help to draw the conjugated antibiotic into the pathogen (de Carvalho and Fernandes, 2014).

Another group of sideromycins known as salmycins consist of an aminoglycoside linked to the tri-hydroxamate siderophore, danoxamine via a succinyl link. These can also be combined with ciprofloxacin to inhibit multidrug resistance in MRSA and MRSE (Sulik et al., 2020). In addition, salmycin and its derivatives are effective agents in preventing bacterial biofilm formation, inhibiting the growth of high-biofilm producers such as *Staphylococcus epidermidis* ATCC 35984 (Sulik et al., 2020).

In some instances daptomycin has been conjugated to an *A*. *baumannii* selective siderophore resulting in potent activity against multidrug resistant strains both *in vitro* and *in vivo*. This is quite unusual because daptomycin is usually only effective against Gram-positive organisms. This study also demonstrates that antibiotics larger than the siderophore can be delivered into the Gram-negative membrane by active transport which overcomes permeability problems (Ghosh et al., 2017).

Even innocuous secondary metabolites such as pigments are now being considered as an aid to antimicrobial activity. For example, a green pigment produced by the marine bacteria *Streptomyces tunisiensis* W4 was reported to have synergistic inhibitory activity against *E*. *faecalis* when combined with cefuroxime and ciprofloxacin (Ibrahim et al., 2023). In some instances, pigments even have antibiotic activity on their own such as undecylprodigiosin, a red pigment produced by *Streptomyces* sp. JAR6 which has been noted for its antibiotic activity against *Salmonella* sp., *Proteus mirabilis*, *Shigella* sp. and *Enterococcus* sp. (Abraham and Chauhan, 2017).

Another secondary metabolite, meridianin was purified from the marine invertebrate *Aplidium meridianum* discovered off the South Georgia Islands. Meridianins consist of a brominated and/or hydroxylated indole framework linked to a 2-aminopyrimidine moiety at C-3 position (Huggins et al., 2018). These metabolites have many biological activities including protein kinase inhibition, adipogenesis inhibition, antitumor activity, and antimalarial activity. Although these secondary metabolites have antimicrobial activity on their own, i.e., inhibition of MRSA, it was recently discovered that they can increase the potency of colistin against colistin resistant and sensitive bacteria (Huggins et al., 2018). This is quite important since colistin is a drug of last resort and also the effective dosage could be lowered reducing the toxicity problems.

**FIGURE 3:**
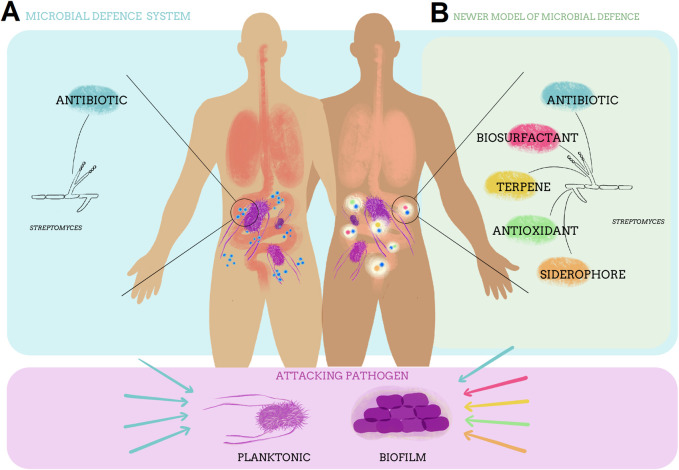
The evolving understanding of bacterial defence and attack systems and their potential utility in creating a more sustainable method of treating pathogenic diseases. **(A)** Represents earlier concepts of a single antimicrobial component which treats a pathogenic disease caused by planktonic bacteria. **(B)** Represents the “newer understandings” of microbial defence which include other secondary metabolites and their potential utility in the treatment of diseases caused by planktonic or biofilm physiologies. Illustration Valentina Romaní Glavich.

Some researchers caution that hybrid combinations of antibiotics will also have the additional burden of increased monitoring, unknown synergies in toxicity or even an increased permeability barrier into Gram-negative organisms (Koh et al., 2023). While this may be true of laboratory derived combinations, pairings that are more similar to natural templates have been successfully approved as “first in class” antibiotics, for example, cefiderocol, which is a conjugation with a siderophore (Wu et al., 2020). In addition, there are many useful pairings of antibiotics and other compatible secondary metabolites that should be examined (Thakur et al., 2024) (Figure 3).

## Adverse events

We cannot mention all the positive advances in antimicrobial technology without cautioning about some of the major obstacles that have to be overcome in their development such as over-prescription, toxicity and the repetition of the discovery process that brought us to this crisis in the first place. Without doubt the discovery of penicillin and streptomycin in the earlier part of the 20th century was one of the greatest advances in medical science. However, even these early discoveries were not without their safety issues (Lobanovska and Pilla, 2017). Fortunately, most safety testing has improved since this time; however it is important that clinicians should be extra cautious about taking shortcuts in the testing and assessment of new antibiotics.

One of the most well-known side-effects of antibiotics is the disruption of gut microbiota or dysbiosis. Numerous studies have found that antibiotic-related disturbances to the gut microbiome increase vulnerability to further infections and are associated with gastrointestinal, kidney, liver, and other problems (Thabet et al., 2024). One potential solution to this problem is to identify an antibiotic that does not harm the microflora of the gut. This is what a research team from the University of Illinois Urbana-Champaign recently achieved. The team discovered a new antibiotic lolamycin (Muñoz et al., 2024) which is a Gram negative-specific antibiotic which targets the protein, LpxH, which is used in a pathway by Gram-negative bacteria to synthesize lipopolysaccharide. This antibiotic was reported to have antimicrobial activity against 130 multidrug-resistant clinical isolates, selectively killing pathogenic Gram-negative bacteria as a consequence of low sequence homology for the target in pathogenic bacteria versus commensals. There is no pre-existing resistance to this class of compounds (Muñoz et al., 2024).

## Conclusion

The over-prescription of antibiotics can be solved by careful stewardship of existing stocks of antibiotics and by identifying and prioritising urgent cases as the WHO have done. Some progress in new antibiotic discovery has been made by exploiting artificial intelligence, exploring new environments for novel antibiotic-producing organisms, and revisiting traditional medicines. Other approaches, such as disguising antibiotics as siderophores, can also contribute to reinvigorating the availability of new antimicrobials. Researchers and health regulators involved in long term solutions to pathogenic diseases should carefully examine whether the approval of new antibiotics, especially those based on known core structures is not just a policy of “kicking the can down the road”. Indeed, as we have highlighted, perhaps they should assess whether a complementary compound approach would be a more sustainable method for reducing the development of antimicrobial resistance. Combination therapies cannot only drastically reduce AMR, but also contribute to combatting biofilms which can otherwise evade treatment.
